# Severe tricuspid regurgitation after mitral valve surgery: the risk factors and results of the aggressive application of prophylactic tricuspid valve repair

**DOI:** 10.1007/s00595-016-1395-4

**Published:** 2016-08-08

**Authors:** Hiroshi Takano, Miyoko Hiramatsu, Hirota Kida, Mitsuru Uenoyama, Kei Horiguchi, Takashi Yamauchi, Keiwa Kin, Yukitoshi Shirakawa, Mitsunori Kaneko, Takashi Daimon

**Affiliations:** 1 Department of Cardiovascular Surgery, Osaka General Medical Center, 3-1-56 Bandaihigashi, Sumiyoshiku, Osaka, 558-8558 Japan; 2 Department of Thoracic and Cardiovascular Surgery, Dokkyo Medical University Koshigaya Hospital, 2-1-50 Minamikoshigaya, Koshigaya, 343-8555 Japan; 3 Department of Cardiovascular Surgery, Suita Tokushukai Hospital, 21-1 Senriokanishi, Suita, 565-0814 Japan; 4 Department of Biostatistics, Hyogo College of Medicine, 1-1 Mukugawacho, Nishinomiya, 663-8501 Japan

**Keywords:** Mitral valve, Surgery, Tricuspid regurgitation, Tricuspid valve repair, Tricuspid annular dilatation

## Abstract

**Purpose:**

This study aimed to examine the risk factors for severe postoperative tricuspid regurgitation (TR) in patients undergoing mitral valve surgery. We also studied the effects of prophylactic tricuspid valve repair (TVR) on severe postoperative TR.

**Methods:**

We retrospectively studied 125 patients without severe TR who underwent mitral valve surgery from 1987 to 2006. Patients did not undergo TVR before 1998 (the early period, *n* = 54). In 1998 (the late period, *n* = 71), patients with a preoperative tricuspid annular diameter of ≥35 mm underwent TVR using an annuloplasty ring (*n* = 52).

**Results:**

In the analysis of the early period, the rates of freedom from severe TR at 10 and 20 years after surgery were 76 and 59 %, respectively. A multivariate analysis identified moderate preoperative TR as a significant risk factor for severe TR. In the late period, none of the 52 patients who underwent TVR developed severe TR. However, 4/19 patients who did not undergo TVR developed severe TR, and all of these four patients had a preoperative tricuspid annular diameter of ≤35 mm.

**Conclusions:**

Moderate preoperative TR is a significant risk factor for severe postoperative TR in patients undergoing mitral valve surgery. The aggressive application of TVR can prevent severe postoperative TR; however, tricuspid annular dilatation might not be a good indicator for TVR.

## Introduction

Severe tricuspid regurgitation (TR) is associated with a reduction in exercise capacity and a poor functional outcome [1, 2]. Patients with severe TR who undergo mitral valve surgery should undergo concomitant tricuspid valve repair (TVR) because TR does not usually resolve if only the mitral valve is repaired. However, it remains unclear whether patients with moderate or mild TR should undergo TVR at the time of mitral valve surgery. Some patients with moderate or mild preoperative TR develop severe late postoperative TR [3–9]. Thus, some authors recommend that such patients should undergo prophylactic TVR at the time of mitral valve surgery [3, 6–10]. The recent guidelines for the management of valvular heart disease from the European Society of Cardiology/European Association for Cardio-thoracic Surgery [11] and the American College of Cardiology/American Heart Association [12] recommend that concomitant TVR should be considered in patients with tricuspid annular dilatation, prior evidence of right heart failure, or pulmonary hypertension (recommendation class IIa or IIb).

We have seen a considerable number of patients with insignificant preoperative TR who developed severe TR after mitral valve surgery. Since 1998, we have therefore performed TVR (tricuspid annuloplasty using a flexible prosthetic ring) more aggressively for patients with a dilated tricuspid annulus, regardless of the severity of TR. In this study, we retrospectively studied the risk factors for the development of severe TR in patients without severe preoperative TR dating back to the period before we began aggressively applying TVR. We also studied the effects of prophylactic TVR on severe postoperative TR. Finally, we assessed the validity of our indication (i.e., tricuspid annular dilatation) for TVR in patients without severe TR who underwent mitral valve surgery.

## Methods

### Study population

**Fig. 1:**
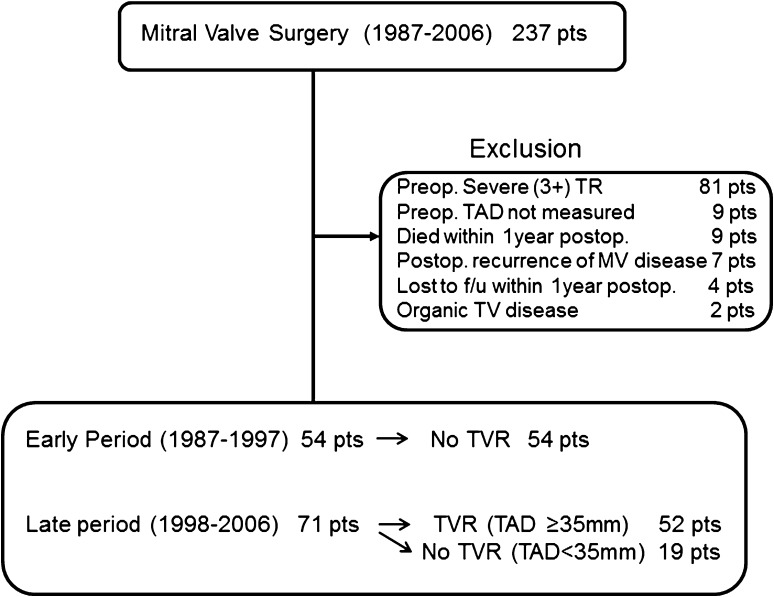
A flow diagram of patients in the study. *pts* patients, *TR* tricuspid regurgitation, *TAD* tricuspid annular diameter, *MV* mitral valve, *f/u* follow-up, *TV* tricuspid valve, *TVR* tricuspid valve repair

Between 1987 and 2006, 237 patients underwent mitral valve surgery at Osaka General Medical Center (Fig. 1). We excluded cases involving severe (grade 3+) preoperative TR (*n* = 81), patients in whom the tricuspid annular diameter (TAD) was not measured (*n* = 9), patients who died within 1 year after surgery (including six hospital deaths) (*n* = 9), patients with the postoperative recurrence of significant mitral valve disease or periprosthetic valvular leakage (*n* = 7), patients who were lost to follow-up within 1 year (*n* = 4), and patients with organic (rheumatic) tricuspid valve disease (*n* = 2). The remaining 125 patients were included in this study. The study protocol was approved by the institutional ethics committee of Osaka General Medical Center (Approval number: 27-S1901).

In 1998, we changed our policy regarding TVR in patients undergoing mitral valve surgery. Before 1998 (early period), we only repaired the tricuspid valve in patients with severe TR. Beginning in 1998 (late period), we considered patients with a TAD of ≥35 mm as candidates for concomitant TVR, regardless of the presence or severity of TR. Thus, none of the 54 patients in the early period of this study underwent TVR, and 52 of the 71 (73 %) patients in the late period underwent TVR. Seven late-period patients were not treated according to the above-mentioned policy at the discretion of the surgeon; these included 6 of the 57 patients with a TAD of ≥35 mm who did not undergo TVR and one patient with a TAD of <35 mm (28 mm) who underwent TVR.

The characteristics of the 125 patients (early period, *n* = 54; late period, *n* = 71) are shown in Table 1. Because there was a time difference between the early and late periods, the characteristics of the two groups, such as age, the rate of rheumatic disease, and the rate of atrial fibrillation or pulmonary hypertension (defined by a mean pulmonary arterial pressure of ≥25 mmHg on cardiac catheterization or preoperative echocardiography), were different from each other. The mitral valve was replaced in the majority of patients in this study, and this is conspicuous in the early period, where all but one underwent mitral valve replacement.

**Table 1:** The baseline characteristics of the patients

Operative period characteristics	All (*n* = 125)	Early period (1987–1997, *n* = 54)	Late period (1998–2006 *n* = 71)	*P* value
Age (years)	58.0 ± 12.0	53.4 ± 11.7	61.5 ± 11.1	<0.001
Age > 50 years	103 (82.4 %)	40 (74.1 %)	63 (88.7 %)	0.06
Male sex	54 (43.2 %)	25 (46.3 %)	29 (40.8 %)	0.59
BSA (m^2^)	1.53 ± 0.17	1.50 ± 0.15	1.55 ± 0.17	0.15
BSA ≥1.50 m^2^	61 (48.8 %)	19 (35.2 %)	42 (59.2 %)	0.008
Rheumatic disease	69 (55.2 %)	39 (72.2 %)	30 (42.3 %)	0.001
Mitral stenosis dominant	56 (44.8 %)	34 (63.0 %)	22 (31.0 %)	0.001
Acute onset of MV disease (<1 month)	10 (8.0 %)	3 (5.6 %)	7 (9.9 %)	0.512
Concomitant aortic valve surgery	44 (35.2 %)	19 (35.2 %)	25 (35.2 %)	1.0
Redo surgery	20 (16.0 %)	11 (20.4 %)	9 (12.7 %)	0.25
Pulmonary hypertension	63 (50.4 %)	36 (66.7 %)	27 (38.0 %)	0.002
Atrial fibrillation	70 (56.0 %)	38 (70.4 %)	32 (45.1 %)	0.005
MV repair	11 (8.8 %)	1 (1.9 %)	10 (14.1 %)	0.02
Maze procedure	6 (4.8 %)	1 (1.9 %)	5 (7.0 %)	0.23
Preoperative TR 0:1+:2+	5:82:38	4:37:13	1:45:25	0.13
TAD (mm)	36.5 ± 5.2	36.0 ± 6.1	36.9 ± 4.3	0.34
TAD ≥35 mm	89 (71.2 %)	32 (59.3 %)	57 (80.3 %)	0.01
Indexed TAD (mm/m^2^)	24.1 ± 3.8	24.1 ± 4.5	24.1 ± 3.2	0.91
Indexed TAD ≥21 mm/m^2^	95 (76.0 %)	36 (66.7 %)	59 (83.1 %)	0.03
LAD (mm)	55.7 ± 10.4	57.0 ± 10.0	54.6 ± 10.6	0.21
LAD ≥60 mm	45 (36.0 %)	22 (40.7 %)	23 (32.4 %)	0.34
Indexed LAD (mm/m^2^)	36.8 ± 7.7	38.3 ± 7.8	35.7 ± 7.5	0.06
Indexed LAD ≥35 mm/m^2^	72 (57.6 %)	36 (66.7 %)	36 (50.7 %)	0.07
LVDd (mm)	55.1 ± 9.1	54.0 ± 10.3	56.0 ± 8.1	0.23
LVDd ≥55 mm	62 (49.6 %)	24 (44.4 %)	38 (49.6 %)	0.32
Indexed LVDd (mm/m^2^)	36.3 ± 6.1	36.1 ± 7.0	36.4 ± 5.3	0.79
Indexed LVDd ≥35 mm/m^2^	63 (50.4 %)	25 (46.3 %)	38 (53.5 %)	0.42
LVDs (mm)	36.2 ± 7.3	36.2 ± 7.9	36.3 ± 6.9	0.93
LVDs ≥40 mm	39 (31.2 %)	20 (37.0 %)	19 (26.8 %)	0.22
Indexed LVDs (mm/m^2^)	23.9 ± 5.0	24.2 ± 5.2	23.7 ± 4.9	0.60
Indexed LVDs ≥25 mm/m^2^	44 (35.2 %)	23 (42.6 %)	21 (29.6 %)	0.13
LVEF	0.66 ± 0.08	0.66 ± 0.08	0.66 ± 0.09	0.91
LVEF <0.65	50 (40.0 %)	24 (44.4 %)	26 (36.6 %)	0.376
Tricuspid valve repair	52 (41.6 %)	0 (0 %)	52 (73.2 %)	<0.001
Follow-up period (years)	12.2 ± 6.0	16.0 ± 6.7	9.5 ± 3.3	<0.001

The data are shown as the mean ± standard deviation or number (frequency)

*BSA* body surface area, *MV* mitral valve, *TR* tricuspid regurgitation, *LAD* left atrial dimension, *LVDd* left ventricular diastolic dimension, *LVDs* left ventricular systolic dimension, *LVEF* left ventricular ejection fraction, *TAD* tricuspid annular diameter

The severity of preoperative TR was classified as none (grade 0) in five patients, mild (grade 1+) in 82, and moderate (grade 2+) in 38. The mean (standard deviation [SD]) TAD measured in late diastole was 36.5 (5.2) mm and the mean (SD) indexed TAD (TAD/body surface area) was 24.1 (3.8) mm/m^2^.

### Surgical procedure

Surgery was performed under moderate hypothermia and cardiopulmonary bypass. Mitral valve surgery and concomitant procedures were performed with aortic cross-clamping and either repeated antegrade or antegrade and retrograde cold cardioplegia. Only one patient in the early period underwent left atrial plication for a huge left atrium.

To repair the tricuspid valve, tricuspid annuloplasty using a flexible ring was performed in 52 patients (all in the late period). A Duran-Medtronic ring (Medtronic, Minneapolis, MN, USA) was used in seven patients and a Cosgrove–Edwards ring (Edwards Life Sciences, Irvine, CA, USA) in 45 patients. With regard to the size of the ring, a 33-mm Duran ring or #30-mm Cosgrove ring was used for male patients, while a #31-mm Duran ring or #28-mm Cosgrove ring was used for female patients. Procedures other than ring annuloplasty, such as edge-to-edge repair, were not performed for the tricuspid valve.

### Echocardiographic measurements

Preoperative transthoracic echocardiography, including two-dimensional echocardiographic and Doppler color flow examinations, was performed in all of the patients by the same experienced operator (M.H.) during the week before surgery.

TR was assessed by Doppler color flow mapping images of the regurgitant jet and pulsed wave Doppler evidence of systolic flow reversal in the inferior vena cava or hepatic veins. The view with maximal spatial distribution of the regurgitant jet was selected, and the jet and right atrial areas were measured in the same frame by planimetry. The severity of TR was graded as follows: none (grade 0), no regurgitant jet; mild (grade 1+), a jet area of <20 % of the right atrial area; moderate (grade 2+), a jet area of 20–33 % of the right atrial area; and severe (grade 3+), a jet area of >33 % of the right atrial area [3, 13, 14]. Patients with severe preoperative TR were excluded from the study. If the estimated ratio of the jet area to the right atrial area was close to a cutoff point, the grade of jet eccentricity was increased to the next higher value. Systolic flow reversal in the inferior vena cava or hepatic veins on pulsed wave Doppler images was considered to indicate at least moderate TR, regardless of the other findings [3, 14].

**Fig. 2:**
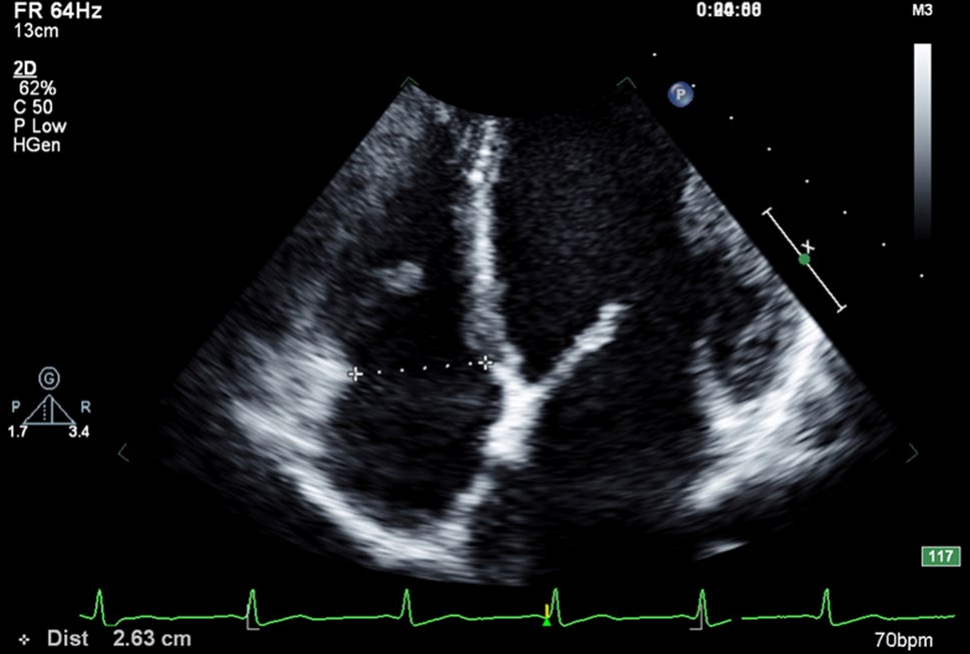
The measurement of the tricuspid annular diameter

In addition to valvular examinations and routine measurements (e.g., left ventricular and left atrial dimensions), the TAD was measured in the apical four-chamber view in late diastole at the time of maximal tricuspid valve opening, as recommended by Foale et al. [15] (Fig. 2).

Postoperative transthoracic echocardiography was performed before discharge and every year thereafter.

### Follow-up

All of the patients were followed up at the Osaka General Medical Center at 3, 6, and 12 months postoperatively. After 1 year, most patients (>90 %) were followed up at 3- or 6-month intervals at the Osaka General Medical Center; the other patients were followed up by local cardiologists. Most patients underwent echocardiography, which was performed by the same experienced operator (M.H.), each year at the Osaka General Medical Center. With the exception of five patients who were lost to follow-up at >5 years after surgery, follow-up is ongoing for all of the surviving patients.

The follow-up for severe TR was censored when the patient died without severe postoperative TR. Although there were three patients whose TR diminished from severe (grade 3+) to moderate (grade 2+), probably due to enhanced medication, the follow-up of their TR was discontinued when severe TR was first observed.

### Statistical analysis

Continuous and categorical variables are reported as the mean and standard deviations (SD), and as frequencies with percentages, respectively. Continuous variables were compared using Student’s *t* test. Categorical variables were compared using Fisher’s exact test if any cell had a count of less than 5 in a contingency table; otherwise, the Chi-squared test was used. Curves for survival and freedom from severe TR were estimated using the Kaplan–Meier method and compared using the log-rank test. A univariate analysis using a log-rank test was used to identify the risk factors for severe TR. A multivariate analysis, which included the risk factors with *P* values of <0.2 in the univariate analysis, was adjusted using the Cox proportional hazard regression model with variable selection (such as forward, backward, and stepwise selection). Hazard ratios and 95 % confidence intervals (CIs) were estimated using the Cox proportional hazard regression model. Changes in the TAD from the preoperative period to the postoperative period were compared between groups by a two-way repeated-measures analysis of variance (ANOVA) that included the main effects of the group and time and the interaction effect between them.

All of the significance tests were two sided and *P* values of <0.05 were considered to indicate statistical significance. All of the statistical analyses were performed using the SPSS 21.0 software program (IBM, Armonk, NY, USA).

## Results

### Survival

The follow-up period ranged from 1.0 to 24.7 years (mean 12.3 years; median 11.7 years). Late death occurred in 38 patients at 1–23 years after surgery. The causes of death included heart failure (*n* = 8), malignancy (*n* = 6), septicemia (*n* = 4), cerebral bleeding (*n* = 3), pneumonia (*n* = 3), renal failure (*n* = 2), cerebral infarction (*n* = 1), and bowel necrosis (*n* = 1); the cause of death was unknown in 10 patients. Six of the 8 patients who died of heart failure had severe TR at the time of death. Five patients were lost to follow-up at 5–18 years.postoperatively. The survival rates at postoperative years 5, 10, 15, and 20 were 91, 81, 64, and 59 %, respectively. The survival of the patients in the early and late periods did not differ to a statistically significant extent (*P* = 0.90).

### The rate of postoperative freedom from severe TR in the earl-period patients

**Fig. 3:**
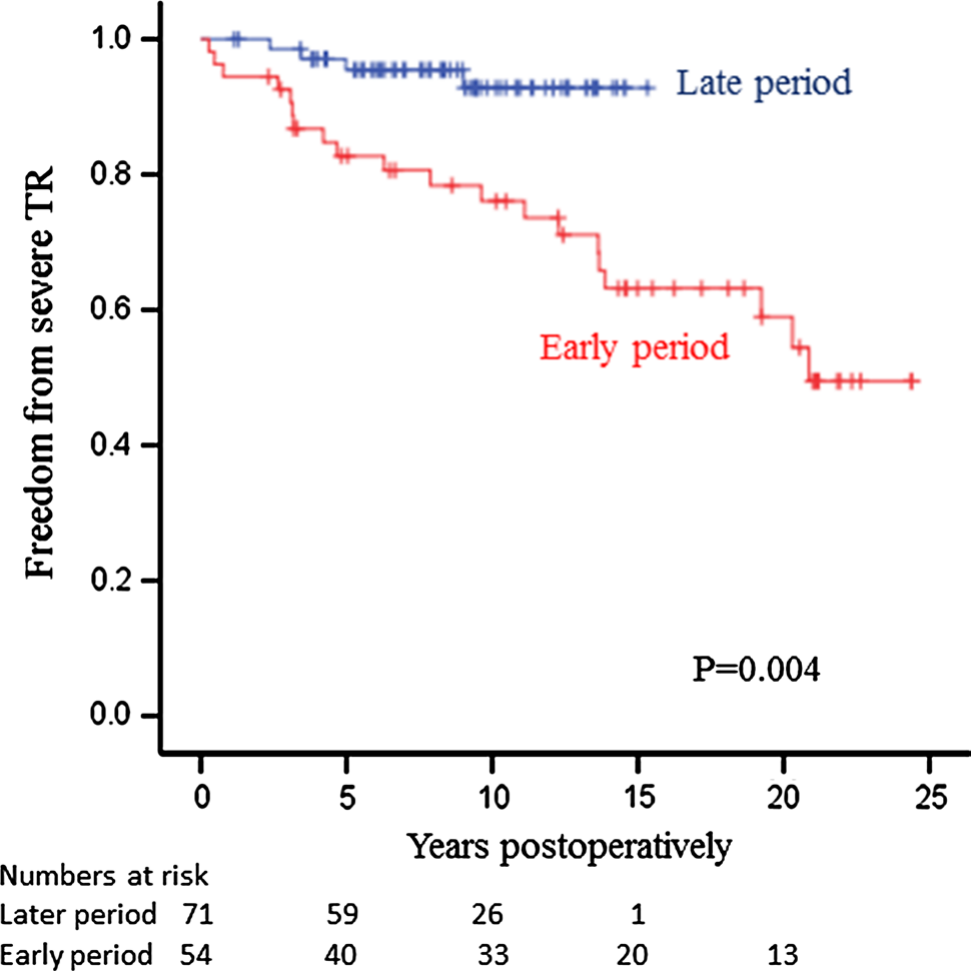
Freedom from severe TR in patients of the early and late periods. *TR* tricuspid regurgitation. *P* = 0.004 (log-rank test)

Nineteen of the 54 early-period patients (none of whom underwent TVR) developed severe postoperative TR. The rates of freedom from severe TR at postoperative years 5, 10, 15, and 20 were 83, 76, 63, and 59 %, respectively (Fig. 3). Three patients in the early period underwent TVR during the follow-up period. All of these three patients underwent aortic valve replacement and tricuspid valve annuloplasty (two for severe TR and one for moderate TR). One of these patients died of septicemia during the perioperative period.

### The risk factors for severe postoperative TR in the early-period patients

**Fig. 4:**
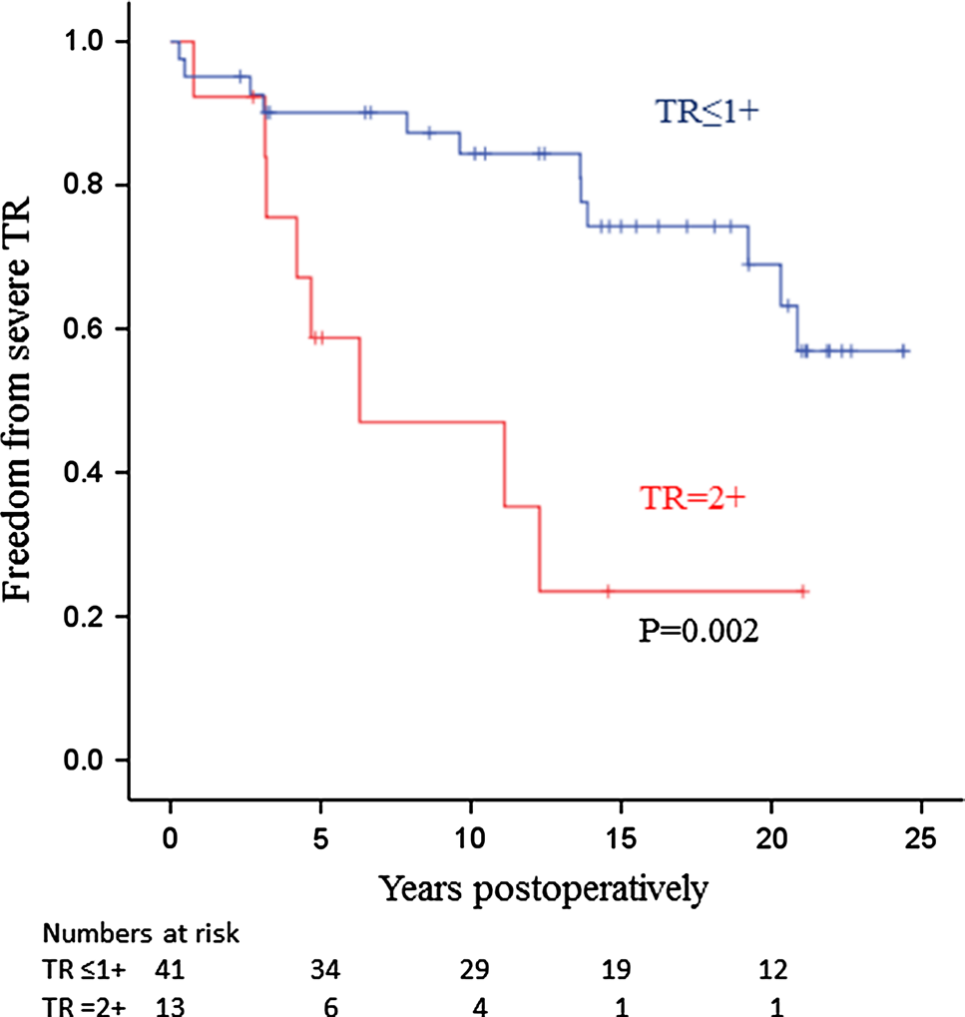
Freedom from severe TR in the early-period patients with grade 2+ and ≤1+ TR. The rate of freedom from severe TR in patients with grade 2+ TR was significantly lower than that in patients with grade ≤1+ TR (*P* = 0.002; log-rank test). *TR* tricuspid regurgitation

A univariate analysis showed that preoperative moderate (grade 2+) TR, atrial fibrillation, an indexed left atrial diameter of ≥35 mm/m^2^, and ≥50 years of age were significantly associated with severe postoperative TR (all *P* < 0.05, log-rank test) (Table 2). Seven variables, including a dominant lesion of mitral stenosis, a body surface area of ≥1.50 m^2^, and a left ventricular systolic dimension of ≥40 mm, in addition to the four significant variables listed above, showed a *P* value of <0.2. These factors were entered into a multivariate analysis using the Cox proportional-hazards model with variable selection methods (forward, backward, and stepwise methods). All of the methods provided the same result: preoperative moderate (grade 2+) TR was a significant risk factor for severe postoperative TR (Table 2). The rates for freedom from severe TR at postoperative years 5, 10, and 15 were 59, 47, and 24 %, respectively, for the 13 patients with grade 2+ TR, and 90, 84, and 74 %, respectively, for the 41 patients with grade ≤1+ TR (Fig. 4).

**Table 2:** The univariate and multivariate analysis of risk factors for severe TR after mitral valve surgery in the early-period patients (1987–1997)

Factors	Number (frequency, %)	Univariate analysis		Multivariate analysis	
		HR (95 % CI)^a^	*P* value^c^	Adjusted HR (95 % CI)^a^	Adjusted *P* value
Age >50 years	40 (74.1)	4.28 (0.99–8.5)	0.034		
Male sex	25 (46.3)	0.71 (0.29–1.75)	0.457		
BSA ≥1.50 m^2^	19 (35.2)	0.50 (0.18–1.39)	0.175		
Rheumatic disease	39 (72.2)	2.15 (0.63–7.37)	0.210		
Mitral stenosis dominant	34 (63.0)	2.27 (0.65–6.79)	0.133		
Acute onset of MV disease (<1 month)	3 (5.6)	NE^b^	0.263		
Concomitant aortic valve surgery	19 (35.2)	1.72 (0.69–4.26)	0.236		
Redo surgery	11 (20.4)	0.89 (0.30–2.68)	0.841		
Pulmonary hypertension	36 (66.7)	1.42 (0.54–3.73)	0.469		
Atrial fibrillation	38 (70.4)	4.91 (1.14–21.21)	0.018	3.97 (0.90–17.5)	0.068
MV repair	1 (1.9)	NE^b^	0.495		
Maze procedure	1 (1.9)	NE^b^	0.495		
Moderate (2+) TR	13 (24.1)	3.89 (1.54–9.84)	0.002	3.08 (1.21–7.82)	0.018
TAD ≥35 mm	32 (59.3)	1.39 (0.57–3.42)	0.470		
Indexed TAD ≥21 mm/m^2^	36 (66.7)	1.48 (0.57–3.86)	0.424		
LAD ≥60 mm	22 (40.7)	1.66 (0.69–4.02)	0.253		
Indexed LAD ≥35 mm/m^2^	36 (66.7)	3.87 (1.13–13.26)	0.020		
LVDd ≥55 mm	24 (44.4)	0.59 (0.24–1.49)	0.262		
Indexed LVDd ≥35 mm/m^2^	25 (46.3)	0.92 (0.38–2.23)	0.854		
LVDs ≥40 mm	20 (37.0)	0.52 (0.19–1.43)	0.197		
Indexed LVDs ≥25 mm/m^2^	23 (42.6)	1.20 (0.50–2.89)	0.691		
LVEF <0.65	24 (44.4)	0.91 (0.37–2.24)	0.843		

*TR* tricuspid regurgitation, *HR* hazard ratio, *BSA* body surface area, *MV* mitral valve, *LAD* left atrial dimension, *LVDd* left ventricular diastolic dimension, *LVDs* left ventricular systolic dimension, *LVEF* left ventricular ejection fraction, *TAD* tricuspid annular diameter, *CI* confidence interval, *NE* not estimable

^a^The HRs and 95 % CIs were estimated using the Cox proportional-hazards model

^b^The numbers of patients with an acute onset of MV disease (<1 month), MV repair, and the maze procedure were small. Thus, the parameters of these factors were not estimable

^c^The *P* values were based on the log-rank test

### Freedom from severe TR in the late-period patients

Four of the late-period patients developed severe TR. The rates for freedom from severe TR at postoperative years 5, 10, and 15 were 96, 93, and 93 %, respectively (Fig. 1). The rate of freedom from severe TR in the late-period patients was significantly higher than that in the early-period patients (*P* = 0.004). One patient in the late period underwent TVR with a prosthetic ring during the follow-up period for severe TR.

**Fig. 5:**
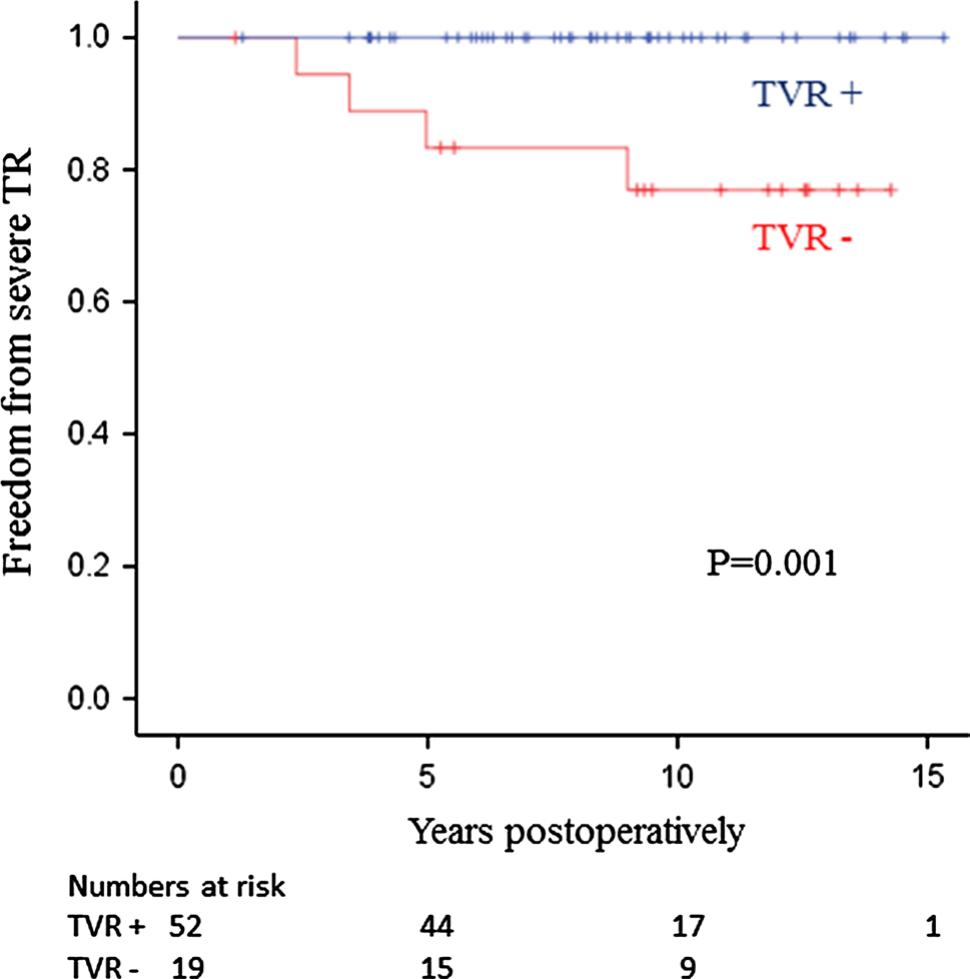
Freedom from severe TR in the late-period patients who underwent TVR and those who did not undergo TVR. None of the patients who underwent TVR developed severe TR. *TR* tricuspid regurgitation, *TVR* tricuspid valve repair. *P* = 0.001 (log-rank test)

Table 3 shows the characteristics of the 52 patients who underwent TVR and the 19 patients who did not in the late period. Besides a larger TAD, the patients who underwent TVR had a greater number of potential risk factors for severe postoperative TR (such as grade 2+ TR, atrial fibrillation, and a greater left atrial dimension) in the early period than the patients who did not undergo TVR. Nonetheless, none of the patients who underwent TVR developed severe postoperative TR. In contrast, four of the 19 patients who did not undergo TVR developed severe postoperative TR. The rates of freedom from severe TR at 5 and 10 years were 100 and 100 %, respectively, in the patients who underwent TVR, and 83 and 77 %, respectively, in the patients who did not undergo TVR (*P* = 0.001, Fig. 5). Table 4 shows the characteristics of each of the four patients who developed severe TR in the late period. The TAD ranged from 27 to 35 mm, and all four patients had a TAD of ≤35 mm. Similarly, the indexed TAD ranged from 17.9 to 22.2 mm, and three of the four patients had an indexed TAD of <21 mm/m^2^.

**Table 3:** The characteristics of patients who did underwent TVR in the late period and those who did not undergo TVR

Characteristics	Underwent TVR (*n* = 52)	Did not undergo TVR (*n* = 19)	*P* value
Age (years)	61.9 ± 9.5	60.3 ± 14.8	0.59
Male sex	19 (36.5 %)	10 (52.6 %)	0.22
Age >50 years	48 (92.3 %)	15 (78.9 %)	0.12
BSA (m^2^)	1.55 ± 0.18	1.52 ± 0.16	0.45
Rheumatic disease	28 (53.8 %)	2 (10.5 %)	0.001
Mitral stenosis dominant	21 (40.4 %)	1 (5.3 %)	0.004
Acute onset of MV disease (<1 month)	3 (5.8 %)	4 (21.1 %)	0.08
Concomitant aortic valve surgery	18 (34.6 %)	7 (36.8 %)	0.87
Redo surgery	8 (15.4 %)	1 (5.3 %)	0.43
Pulmonary hypertension	25 (48.1 %)	2 (10.5 %)	0.005
Atrial fibrillation	30 (57.7 %)	2 (10.5 %)	<0.001
MV repair	4 (7.7 %)	6 (31.6 %)	0.01
Maze procedure	5 (9.6 %)	0 (0 %)	0.32
Preoperative TR 0:1+:2+	0:27:25	1:18:0	<0.001
TAD (mm)	38.5 ± 3.0	32.5 ± 4.3	<0.001
TAD ≥35 mm	51 (98.1 %)	6 (31.6 %)	<0.001
Indexed TAD (mm/m^2^)	25.0 ± 2.8	21.5 ± 2.9	<0.001
Indexed TAD ≥21 mm/m^2^	49 (94.2 %)	10 (52.6 %)	<0.001
LAD (mm)	56.9 ± 9.6	48.4 ± 10.8	0.002
LAD ≥60 mm	21 (40.4 %)	2 (10.5 %)	0.022
Indexed LAD (mm/m^2^)	37.1 ± 7.2	32.0 ± 7.1	0.011
Indexed LAD >35 mm/m^2^	30 (57.7 %)	6 (31.6 %)	0.05
LVDd (mm)	55.5 ± 8.9	57.2 ± 5.4	0.43
Indexed LVDd (mm/m^2^)	35.9 ± 5.5	37.9 ± 4.7	0.15
LVDs (mm)	36.2 ± 7.3	36.4 ± 5.7	0.91
Indexed LVDs (mm/m^2^)	23.5 ± 5.1	24.2 ± 4.5	0.61
LVEF	0.65 ± 0.08	0.69 ± 0.10	0.13

*TVR* tricuspid valve repair, *BSA* body surface area, *MV* mitral valve, *LAD* left atrial dimension, *LVDd* left ventricular diastolic dimension, *LVDs* left ventricular systolic dimension, *LVEF* left ventricular ejection fraction, *TAD* tricuspid annular diameter

**Table 4:** The characteristics of the patients who developed severe postoperative TR in the late period

Pt	Age (years)	Etiology of MV disease	MS or MR	TR	Cardiac rhythm	PH	LAD (mm)	Indexed LAD (mm/m^2^)	TAD (mm)	Indexed TAD (mm/m^2^)	MV repair or replacement	TVR	Time to severe TR (y)
1	56	Rheumatic	MR	1+	AF	No	75	44.6	30	17.9	Replacement	No	9.0
2	53	IE	MR	0	SR	No	40	28.6	27	19.3	Replacement	No	2.4
3	70	IE	MR	1+	SR	Yes	35	21.6	33	20.4	Replacement	No	3.4
4	77	Degenerative	MR	1+	SR	No	54	34.2	35	22.2	Repair	No	5.0

*TR* tricuspid regurgitation, *Pt* patient, *MV* mitral valve, *MS* mitral stenosis, *MR* mitral regurgitation, *PH* pulmonary hypertension, *LAD* left atrial dimension, *TAD* tricuspid annular diameter, *TVR* tricuspid valve repair, *IE* infectious endocarditis, *AF* atrial fibrillation, *SR* sinus rhythm

### Serial changes in the TAD

Because the preoperative and postoperative TAD values were serially measured in most patients, the preoperative and postoperative indexed TAD values were compared among three groups of patients, who were categorized as follows. Group 1 included patients who did not undergo TVR and who did not develop severe postoperative TR (*n* = 39; early period, *n* = 24; late period, *n* = 15). Group 2 included patients who did not undergo TVR and who developed severe postoperative TR (*n* = 18; early period, *n* = 14; late period *n* = 4). Group 3 included patients who underwent TVR (none of them developed severe postoperative TR) (*n* = 51, all in the late period). Seventeen patients in whom the postoperative TAD was not serially measured were excluded from this analysis. In groups 1 and 3, the value that was used for the postoperative TAD was obtained at the last follow-up examination (group 1: postoperative year, 13.7 ± 6.0; group 3: postoperative year, 8.2 ± 3.6). In group 2, the value that was used for the postoperative TAD was obtained at the time of the diagnosis of severe postoperative TR (postoperative year, 9.0 ± 6.2).

**Fig. 6:**
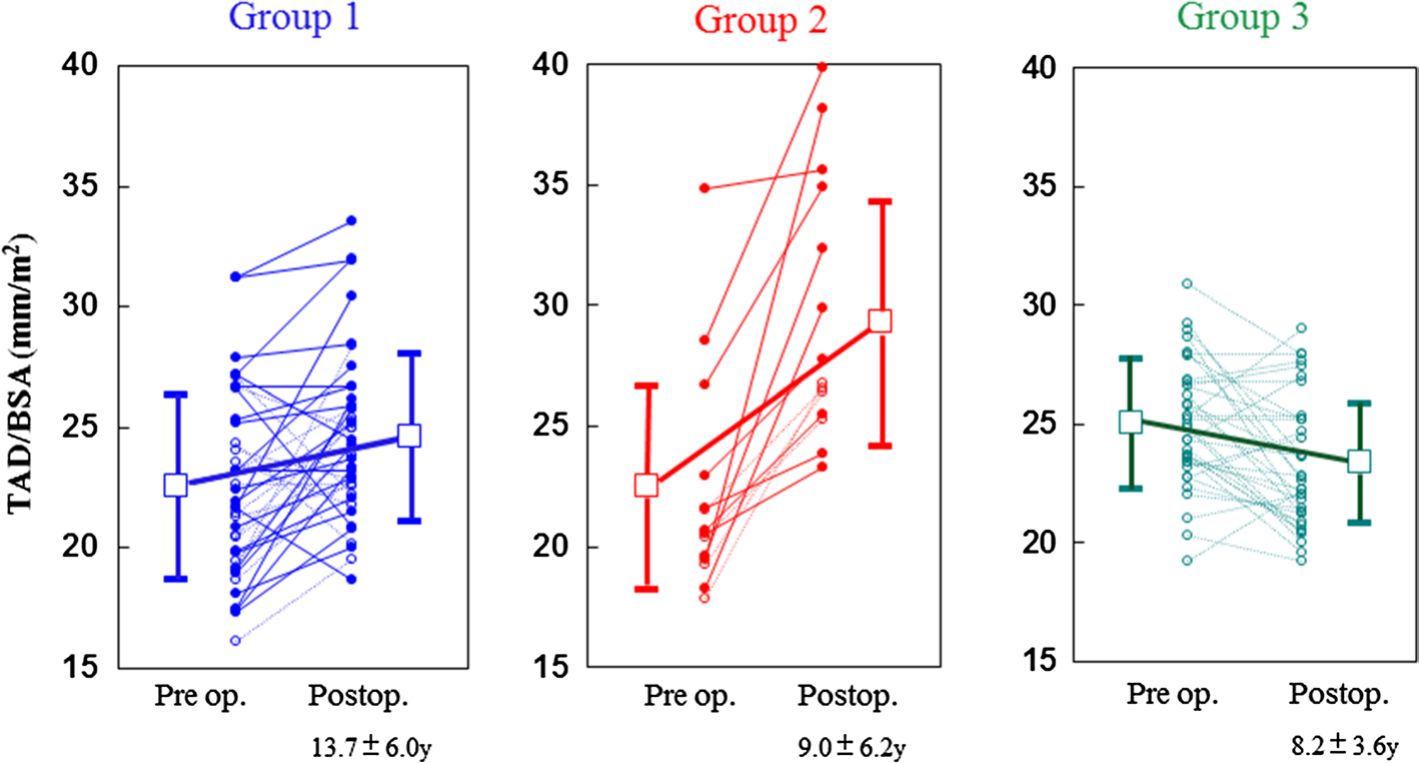
Changes in the indexed tricuspid annular diameter before and after surgery. Group 1 consisted of 39 patients who did not undergo TVR and who did not develop severe postoperative TR. Group 2 consisted of 18 patients who did not undergo tricuspid valve surgery and who developed severe postoperative TR. Group 3 consisted of 51 patients who underwent TVR (none of them developed severe postoperative TR). *Closed circles* indicate the early-period patients; *open circles* indicate the late-period patients. *Open squares* and *bars* indicate the means and standard deviations for each group of patients. A two-way repeated ANOVA showed that the *P* values for the main effects of group and time were *P* = 0.03 and *P* < 0.001, respectively; while the *P* value for the interaction effect between them was *P* < 0.001. See the text for more information about the comparisons between the groups. *BSA* body surface area, *TR* tricuspid regurgitation, *TVR* tricuspid valve repair, *TAD* tricuspid annular diameter

The changes in the indexed TAD values before and after surgery differed among the groups (*P* < 0.001 for an interaction effect between group and time in a two-way repeated-measures ANOVA). Specifically, the preoperative indexed TAD values of the patients in group 3 were larger than those in groups 1 and 2 because TVR was performed in patients with a large TAD. The indexed TAD was increased after surgery in groups 1 and 2, but was decreased after surgery in group 3. The postoperative indexed TAD values of the patients in group 2 were larger than those in groups 1 and 3 (Fig. 6).

## Discussion

The findings of the present study were as follows. First, a relatively high rate of patients without severe preoperative TR developed severe TR after mitral valve surgery if the tricuspid valve was left untouched. Additionally, moderate preoperative TR was the most significant risk factor for severe postoperative TR. Second, the aggressive application of TVR using a prosthetic ring prevented the development of severe postoperative TR for a long period of time. Third, our indication, tricuspid annular dilatation as measured by a preoperative echocardiogram, might not be a good indicator of whether patients undergoing mitral valve surgery should undergo concomitant TVR.

### The prevalence and predictors of significant postoperative TR after mitral valve surgery

Significant TR has previously been reported to develop at a relatively high number of cases after mitral valve surgery [3, 5, 8, 9, 16, 17]. The risk factors for postoperative TR in these studies included older age [3, 17], the grade of preoperative TR [8, 17], atrial fibrillation [8, 9, 16], a huge left atrium [8, 9], a long time from the onset of mitral valve disease to surgery [9], rheumatic changes of the tricuspid valve [9], and a low ejection fraction [16]. In the present study, the rate of severe postoperative TR at 20 years among the early-period patients (none of whom underwent TVR) was 41 %. The univariate analysis revealed that the predictors of severe TR were moderate preoperative TR, a large left atrial dimension, atrial fibrillation, and older age. Our results are therefore similar to those of previous studies.

### The effectiveness of tricuspid valve repair with a prosthetic ring for less than severe TR in patients who underwent mitral valve surgery

In the late period of this study, we aggressively applied TVR using a prosthetic ring in 52 of the 71 patients who underwent mitral valve surgery. Although our indication for TVR was tricuspid annular dilatation (TAD ≥35 mm), these patients also had other risk factors, such as moderate TR, atrial fibrillation, rheumatic disease, pulmonary hypertension, and a large left atrium (Table 3). Nonetheless, the rate of freedom from severe TR was 100 % at 15 years. Thus, prophylactic TVR using a prosthetic ring appears to be effective in preventing the development of severe postoperative TR in patients without severe preoperative TR.

### The validity of tricuspid annular dilatation as an indication for TVR

**Fig. 7:**
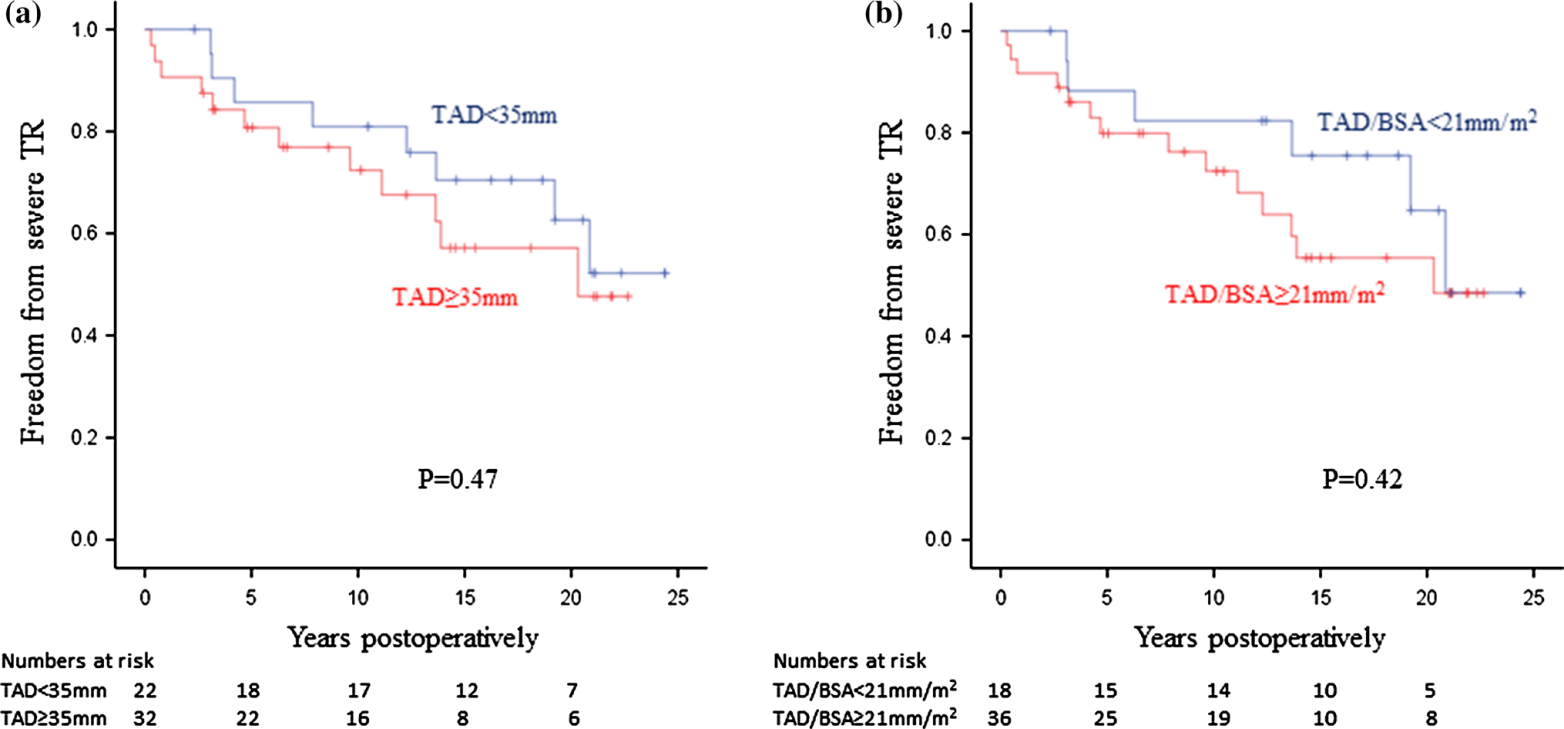
Freedom from severe TR in the early-period patients according to the tricuspid annular diameter. The log-rank test did not show a significant difference between 32 patients with a TAD of ≥35 mm and 22 patients with a TAD of <35 mm (**a**). Furthermore, there was no difference between 36 patients with an indexed TAD of ≥21 mm/m^2^ and 18 patients with an indexed TAD of <21 mm/m^2^ (**b**). *TR* tricuspid regurgitation, *BSA* body surface area, *TAD* tricuspid annular diameter

In the present study, we attempted to examine the validity of our indication (preoperative tricuspid annular dilatation) for prophylactic TVR in patients undergoing mitral valve surgery. In the early-period patients (none of whom underwent TVR), tricuspid annular dilatation (a TAD of ≥35 mm or an indexed TAD of ≥21 mm/m^2^) was not a risk factor for severe postoperative TR (Table 2). The log-rank test did not show a significant difference in the rate of freedom from severe postoperative TR between patients with large and small TAD values (Fig. 7a, b). Furthermore, in the late-period patients in whom tricuspid annular dilatation (TAD ≥35 mm) was used as an indicator for TVR, the cumulative incidence of severe TR at 10 years in patients who did not undergo tricuspid repair was 23 %. We are therefore of the opinion that tricuspid annular dilatation might not be a valid indicator of whether patients undergoing mitral valve surgery should undergo concomitant TVR. This conclusion may be surprising because many surgeons believe that tricuspid annular dilatation is an indicator of TVR in patients undergoing mitral valve surgery, because the guidelines for the management of valvular heart disease suggest that the performance of TVR in such patients.

Tricuspid annular dilatation, as measured by preoperative or intraoperative echocardiography, has been recommended as an indication for TVR by several authors [13, 18, 19] based on the observation that the TAD was correlated with the severity of TR. Following this recommendation, tricuspid annular dilatation was used as an indication for TVR at the time of mitral valve surgery by Tager et al. [20] (TAD ≥30 mm), Colombo et al. [21] (indexed TAD ≥21 mm/m^2^), Van de Veire et al. [22] (TAD ≥40 mm), and Calafiore et al. [23] (systolic TAD ≥24 mm). Dreyfus et al. [6] performed TVR in patients with a TAD of ≥70 mm, which was measured intraoperatively from the anteroseptal to the anteroposterior commissures in the arrested heart. These authors concluded that tricuspid annular dilatation was an appropriate indicator of whether patients undergoing mitral valve surgery should undergo concomitant TVR. However, a detailed examination of the data from the above-mentioned studies might not fully support this conclusion. In the study by Dreyfus et al. [6], 55 out of 163 patients with a TAD of <70 mm developed grade 3+ to 4+ TR at a mean follow-up time of 4.8 years. Moreover, the follow-up period in some of the studies appears to be insufficient [22, 23].

In contrast, other studies have reported that preoperative tricuspid annular dilatation is not a pivotal factor for the progression of TR after mitral valve surgery [24, 25]. Goldstone et al. [17] recently reported that older age and the grade of preoperative TR were independent predictors of the postoperative progression of TR in patients undergoing mitral valve repair for mitral valve prolapse with TR that was assessed as being less than severe. However, they reported that when limited to patients with mild TR (or those without TR), the indexed TAD was the only significant risk factor.

We also analyzed the risk factors of 41 patients in the early period with mild or none (grade 1+ or 0) preoperative TR. We found that atrial fibrillation and a huge left atrium (left atrial dimension ≥35 mm) were significant risk factors for severe postoperative TR, but that tricuspid annular dilatation (a TAD of ≥35 mm or an indexed TAD of ≥21 mm/m^2^) was not a risk factor in a univariate analysis (log-rank test). Because of the small number of patients, our study does not have sufficient statistical power to prove that tricuspid annular dilatation is not a valid indicator of tricuspid valve surgery. We believe that the validity of tricuspid annular dilatation as an indicator of whether patients should undergo TVR needs to be re-evaluated.

### The mechanisms underlying secondary TR after mitral valve surgery

An understanding of the mechanisms underlying secondary TR is improving based on the results of the detailed analysis of the structure of the tricuspid valve. Tricuspid annular dilatation is commonly observed in patients with severe TR [14, 26–28]. We also showed that the TAD was significantly increased in patients who developed severe postoperative TR at the time of its development (Fig. 6).

In addition, the tethering of the tricuspid valve leaflets may play a major role in the development of secondary TR. Right ventricular enlargement due to mitral valve disease causes tricuspid annular dilatation and the displacement of the papillary muscles, leading to the tethering of the tricuspid valve leaflets. This results in inadequate leaflet coaptation [29, 30]. A recent study used three-dimensional echocardiography to evaluate patients with secondary TR and reported that the tricuspid annulus was not only dilated, but also flattened and circular in patients with secondary TR [31].

However, the reason why these changes in the tricuspid valve structure occur after mitral valve surgery is not clear. In the present study, we excluded patients with recurrence or in which the mitral valve lesions were insufficiently repaired and those who developed abnormal prosthetic valve function [32]. Thus, the tricuspid valve was unlikely to have been affected by residual mitral valve disease. The tricuspid valve was also unlikely to have been affected by persistent pulmonary hypertension after mitral valve surgery. This hypothesis is based on our finding that postoperative pulmonary hypertension, as assessed by follow-up echocardiography, was not a significant risk factor for severe postoperative TR in our univariate analysis [*P* = 0.39 (log-rank test); hazard ratio = 1.48, *P* = 0.40 (Cox hazards model)]. Previous studies [1, 6] have also reported similar findings.

Some authors have hypothesized that tricuspid annular dilatation may be an ongoing disease process that continues even after the mitral valve lesion has been repaired [6]. This hypothesis may explain the findings of previous studies, which noted that the incidence of postoperative TR was higher in patients with rheumatic mitral disease than in those with degenerative mitral disease [24]. Additionally, a long time from the onset of mitral valve disease to surgery is a predictor of late TR after mitral valve surgery [9]. However, the hypothesis that tricuspid annular dilatation is an ongoing process is speculative and should be the subject of further studies.

### Study limitations

The present study is associated with several limitations. First, this study was a retrospective analysis with a small sample size. Although our policy of tricuspid valve surgery was consistent during the study period, many surgeons were involved over a relatively long study period. A prospective study with a large number of patients would help to determine the risk factors for postoperative TR, as well as the importance of tricuspid annular dilatation. Second, over half of the patients had rheumatic heart disease, and the mitral valve was replaced in almost all of the patients. Although we accounted for these factors (e.g., rheumatic disease, mitral valve repair, the acute onset of mitral valve disease) in the analysis, our results may not be applicable to patients who undergo mitral valve repair for degenerative disease. Third, the severity of TR was mainly determined by comparing the regurgitant jet area with the right atrial area, rather than by the recommendations of the American Society of Echocardiography [33]. This is because most of the patients in this study underwent surgery before the recommendation had been reported. Finally, we did not evaluate the tethering of the tricuspid valve leaflets, the dimensions and functions of the right ventricle and atrium, or the shape of the tricuspid annulus. These details of the tricuspid valve complex should be further evaluated because they may help to predict secondary TR after mitral valve surgery.

## Conclusions

Relatively high rates of patients without severe preoperative TR develop severe postoperative TR if the tricuspid valve is left untouched at the time of mitral valve surgery. Moderate preoperative TR is the most significant risk factor for severe postoperative TR. The aggressive application of TVR using a prosthetic ring may prevent the development of severe postoperative TR. The measurement of tricuspid annular dilatation by preoperative echocardiography might not be a good indicator of whether patients undergoing mitral valve surgery should undergo concomitant TVR. However, further study will be needed to confirm our results.
